# Strengthening biomedical engineering systems in sub-Saharan Africa: a repeated cross-sectional assessment of workshop readiness and medical device maintenance in NEST360-supported hospitals

**DOI:** 10.7189/jogh.16.04270

**Published:** 2026-09-25

**Authors:** John Wainaina, Christine Alexandra Bohne, George Banda, Vincent Ochieng, Charles Osuagwu, Elizabeth Ngowi, Hope David Peter, Georgia Jenkins, June Madete, Millicent Alooh, Annika Nambiar, Sara Liaghati, Morris Ogero, Rebecca Eliana Penzias, Danica Kumara, Lisa Ruth Hirschhorn, Zillah Maria Oden, Rebecca Richards-Kortum, NEST360 Health Facility Assessment Collaborative Group, Morris Ondieki, Samuel K Ngwala, Evelyn Zimba, Msandeni Chiume, Joseph Misyenje, Lucas Malla, Eric Ohuma, Josephine Shabani, Irabi Kassim, Prosper Mshana, Jacqueline Minja, Nahya Salim, Franklin Okech, David Gathara, Olabisi Dosunmu, Opeyemi Odedere, James H Cross, Edith Gicheha

**Affiliations:** 1 Rice360 Institute for Global Health Technologies, Rice University, Texas, USA; 2 Ifakara Health Institute, Dar es salaam, Tanzania; 3 Kamuzu University of Health Sciences, University of Malawi, Blantyre, Malawi; 4 APIN Public Health Initiatives, Abuja, Nigeria; 5 Kenyatta University, Nairobi, Kenya; 6 UNICEF, Blantyre, Malawi; 7 Centre for Maternal, Adolescent, Reproductive, & Child Health, London School of Hygiene & Tropical Medicine, London, UK; 8 Independent consultant, Washington, D.C., USA; 9 Feinberg School of Medicine, Northwestern University, Chicago, USA

**Keywords:** biomedical engineering, health system readiness, medical device maintenance, low- and middle-income countries, neonatal care, health facility assessment

## Abstract

**Background:**

Biomedical engineering technician (BMET) workshops are essential for maintaining medical devices, yet their service readiness in low-resource settings remains insufficiently described. This study assesses BMET workshop readiness and associated maintenance systems across Newborn Essential Solutions & Technologies 360-supported hospitals in four sub-Saharan African countries.

**Methods:**

We conducted repeated cross-sectional health facility assessments of BMET workshops in 2020–2021 (n = 53 hospitals) and 2023 (n = 63 hospitals) across Kenya, Malawi, Nigeria, and Tanzania. We assessed BMET workshop readiness using 186 indicators across six domains: tools and test equipment, spare parts, storage and organisation, systems and processes, human resources, and maintenance and repair capacity. We scored indicators as 1 (available), 0.5 (partially available), or 0 (not available), and aggregated as percentages at hospital level. We summarised domain and overall readiness using medians (Mdn) and interquartile ranges (IQRs). We assessed differences between samples using Wilcoxon rank-sum tests, and explored associations between readiness, planned preventive maintenance (PPM), and device functionality.

**Results:**

Overall BMET workshop readiness increased from a median of 28.0% (IQR = 20.9–35.8) to 50.0% (IQR = 36.5–56.5) in 2023 (Wilcoxon rank-sum *P* < 0.001). We observed improvements across all domains, with the largest gains in tools and test equipment and maintenance and repair capacity, while human resources showed minimal change. Spare parts availability remained low despite improvement, increasing from 4.0% to 14.8%. PPM system presence increased from 68.5% to 90.6%, although implementation varied. Associations between BMET readiness and device functionality were present at baseline but not in 2023.

**Conclusions:**

BMET workshop readiness improved across participating hospitals between baseline and 2023, particularly in tools and maintenance systems. However, persistent gaps remain in human resources, spare parts availability, and maintenance system implementation. These findings highlight the need for sustained investment in biomedical engineering workforce capacity, supply chain integration, and system strengthening to support durable improvements in medical device functionality.

Functional medical devices are essential for delivering quality care in hospitals. Malfunctioning equipment can lead to delayed treatments, poor patient outcomes, wasted resources, and the proliferation of ‘medical equipment graveyards’ – stockpiles of broken, unused devices [1,2]. Proper maintenance of medical equipment is essential to minimise breakdowns and extend their lifespan [3,4]. Hospital-based biomedical engineering technician (BMET) staff must conduct regular planned preventive maintenance (PPM) and timely corrective maintenance (CM) activities to ensure medical devices function optimally for patient care. This requires appropriately resourced workshops that provide the necessary space, tools, and spare parts to maintain, repair, and calibrate medical equipment [2].

However, in low-resource settings, up to 40% to 70% of medical devices are reported as nonfunctional, underutilised, or never maintained due to persistent challenges faced by BMET departments, including shortages of skilled personnel and a lack of essential tools and spare parts for repairs [2,5]. Additionally, structural challenges such as unreliable power supply, inadequate space, chronic underinvestment in maintenance, and the inappropriate design of medical equipment for these settings hinder equipment availability and functionality [1]. Adding to these challenges, a large fraction of medical devices in low-resource settings are obtained through donations or as commercially available models intended for high-resource environments, often making them difficult to maintain and repair [1].

Many devices are too expensive and/or ill-suited to harsh conditions typical of low-resource settings, including dust, humidity, temperature fluctuations, unstable electrical voltages, and frequent user turnover [1,6]. As a result, frequent breakdowns and poor device functionality lead to prolonged equipment downtime, reducing the availability of essential medical devices needed for patient care [7]. The absence of service and operator manuals and effective health technology management systems further exacerbates these issues [5].

BMET workshops are dedicated technical spaces within hospitals where medical equipment is maintained, repaired, and calibrated, playing a vital role in ensuring equipment functionality and safety. While numerous studies have examined the functionality of medical equipment in clinical settings, assessments of BMET workshop readiness (a composite measure of structural, human resource, equipment, and system capacity within hospital biomedical engineering workshops), especially in resource-limited contexts, remain scarce [8]. Hospital-level service readiness is commonly evaluated using tools such as the Service Availability and Readiness Assessment [8], yet these frameworks often overlook the operational infrastructure provided by BMET workshops. Given the central role of medical devices in healthcare delivery, this gap in understanding workshop readiness may compromise efforts to ensure consistent, high-quality care.

Evidence on BMET workshop readiness at scale remains sparse, and multi-country programme implementations such as Newborn Essential Solutions and Technologies (NEST360) offer a valuable opportunity to document conditions across hospitals in different country contexts. Here, we aimed to describe the BMET workshop readiness levels in NEST360 partner hospitals in Kenya, Tanzania, Malawi, and Nigeria at the baseline (2020–2021) and follow-up (2023); assess differences in BMET workshop readiness between baseline and 2023, recognising differences in hospital samples across time points; and examine the association between BMET workshop readiness scores and PPM activities in partner hospitals.

## METHODS

### NEST360 Alliance

NEST360 is a multi-country consortium working with Ministries of Health in Kenya, Malawi, Nigeria, and Tanzania to strengthen newborn care through integrated technology, training, and health system strengthening interventions [9]. Since 2019, the programme has supported World Health Organization (WHO) Level 2+ neonatal care through a bundled package of essential medical devices (including continuous positive airway pressure (CPAP)), biomedical engineering capacity strengthening, and clinical training [9–11].

The intervention includes procurement and deployment of newborn care equipment, training of clinicians in safe device use, and strengthening of biomedical engineering technician (BMET) workshops through training in installation, calibration, preventive maintenance, and repair. The programme also supported development of national biomedical engineering curricula, hospital maintenance systems, and expanded training across 63 hospitals during Phase 1 (2019–2024) (Kenya n = 13, Malawi n = 36, Nigeria n = 7, Tanzania n = 7). Routine hospital data on service delivery, device functionality, and maintenance practices were also generated to support monitoring and quality improvement.

### Health facility assessment tool development and data collection

The health facility assessment (HFA) tool, designed to assess service readiness for small and sick newborn care (SSNC), was co-developed by NEST360 and UNICEF with global and country-level experts [12]. It was built on existing service readiness tools, norms, and standards, and refined through implementation in the four NEST360 countries [12,13].

The HFA was conducted between September 2019 and March 2021 across NEST360-supported hospitals. The BMET workshop module, part of the larger HFA tool, was introduced in 2020 and therefore was not applied in hospitals before its introduction. As a result, BMET workshop baseline data are available for 53 hospitals assessed between 2020 and 2021. By 2023, follow-up assessments included 63 hospitals. Data were collected using a mobile-based Research Electronic Data Capture (REDCap) application on Android tablets [14], with full procedures described elsewhere [12,13].

This study uses a repeated cross-sectional design to compare the 2020–2021 and 2023 BMET workshops, reflecting the evolving composition of NEST360-supported hospitals.

### BMET module categories

The BMET workshop assessment module covered six categories: tools, spare parts, storage and layout, systems and processes, human resources, and maintenance and repair (Table S1 in the **Online Supplementary Document**). We adapted assessment indicators primarily from the WHO Medical Devices technical series [15] and the Ziken International 6 Guide [16], both widely used resources that provide practical guidance for medical device management, maintenance, and workshop organisation in low-resource settings. In this study, we evaluated these categories through 186 indicators to assess the BMET workshop's capacity to support the maintenance and repair of medical devices, which is essential for delivering SSNC. We calculated the BMET workshop readiness score as an unweighted aggregate of item-level indicators across domains, reflecting the proportion of available items relative to those assessed.

### Scoring methodology and analysis

We scored each indicator as 1 (available), 0.5 (partially available, defined at item level based on predefined criteria such as intermittent availability, or incomplete documentation), or 0 (unavailable). We excluded ‘not applicable’ items from denominators.

For each hospital, we summed item scores within each domain and expressed them as a proportion of applicable items within that domain. We calculated the overall hospital readiness score as the proportion of all applicable item scores aggregated across all domains. Thus, domain scores reflect within-domain availability, whereas the overall score reflects availability across all indicators.

We summarised hospital-level scores at country and programme level using means (x̄) with 95% confidence intervals (CIs), or with medians (Mdn) and interquartile ranges (IQRs) reported for skewed distributions. We assessed differences between baseline and follow-up hospital-level scores using non-parametric tests (Wilcoxon rank-sum test) at a 5% significance level.

Denominators varied by indicator and time point because of differences in missing data; we performed no imputation. To evaluate the robustness of our estimates, we performed sensitivity analyses using a restricted paired data set (hospitals with data at both time points) (**Online Supplementary Document**). We conducted all analyses in *R*, version 4.3.3 (R Core Team, Vienna, Austria).

### PPM actions

Hospital biomedical technicians reported weekly on whether PPM activities were performed for six commonly available device categories (glucometers, pulse oximeters, CPAP machines, oxygen concentrators, radiant warmers, phototherapy machines) using REDCap-based event logs. We aggregated data monthly to determine whether PPM was completed at least once per device category. We defined PPM completeness as the proportion of expected monthly actions completed.

While PPM actions refer to individual maintenance activities recorded through weekly logs and aggregated over time, a PPM system refers to the presence of a documented institutional process for planned preventive maintenance within a hospital.

We conducted correlation analyses using 2023 BMET workshop readiness scores, as PPM reporting began in 2022, after baseline assessments. We summarised PPM data over two periods: 2022 and 2023–2024, to reflect early and later programme implementation phases. These periods were not temporally aligned with baseline readiness assessments, and analyses are therefore interpreted as cross-sectional associations at the hospital level rather than longitudinal comparisons.

For descriptive purposes, we pragmatically categorised readiness scores as very low (0–39%), low (40–59%), moderate (60–79%), or high (≥80%).

### Device availability and functionality assessment

We documented the number of available and functional devices in each hospital’s neonatal unit during each HFA round through direct observation and verification with hospital staff. We defined functionality as devices reported and observed to be operational at the time of assessment. We calculated functionality rates as the proportion of functional devices among all available devices in the neonatal unit at each assessment.

We conducted hospital-level correlation analyses to assess the association between BMET workshop readiness scores and neonatal unit device functionality rates. We interpreted these as cross-sectional associations, recognising that functionality was measured within neonatal units whereas readiness reflects broader workshop capacity at the hospital level. We made no adjustments for device type or hospital level.

## RESULTS

Baseline 2020–2021 BMET workshop data were available for 53 hospitals (Kenya n = 11, Malawi n = 28, Nigeria n = 7, Tanzania n = 7), while 2023 follow-up data were available for 63 hospitals (Kenya n = 13, Malawi n = 36, Nigeria n = 7, Tanzania n = 7). We therefore analysed independent cross-sectional samples at each time point and compared hospital-level readiness scores between periods using non-parametric methods. We derived BMET workshop readiness from 186 indicators across six domains and aggregated these into hospital-level composite scores (Tables S1 and S2 in the **Online Supplementary Document**).

### Overall readiness scores

**Figure 1:**
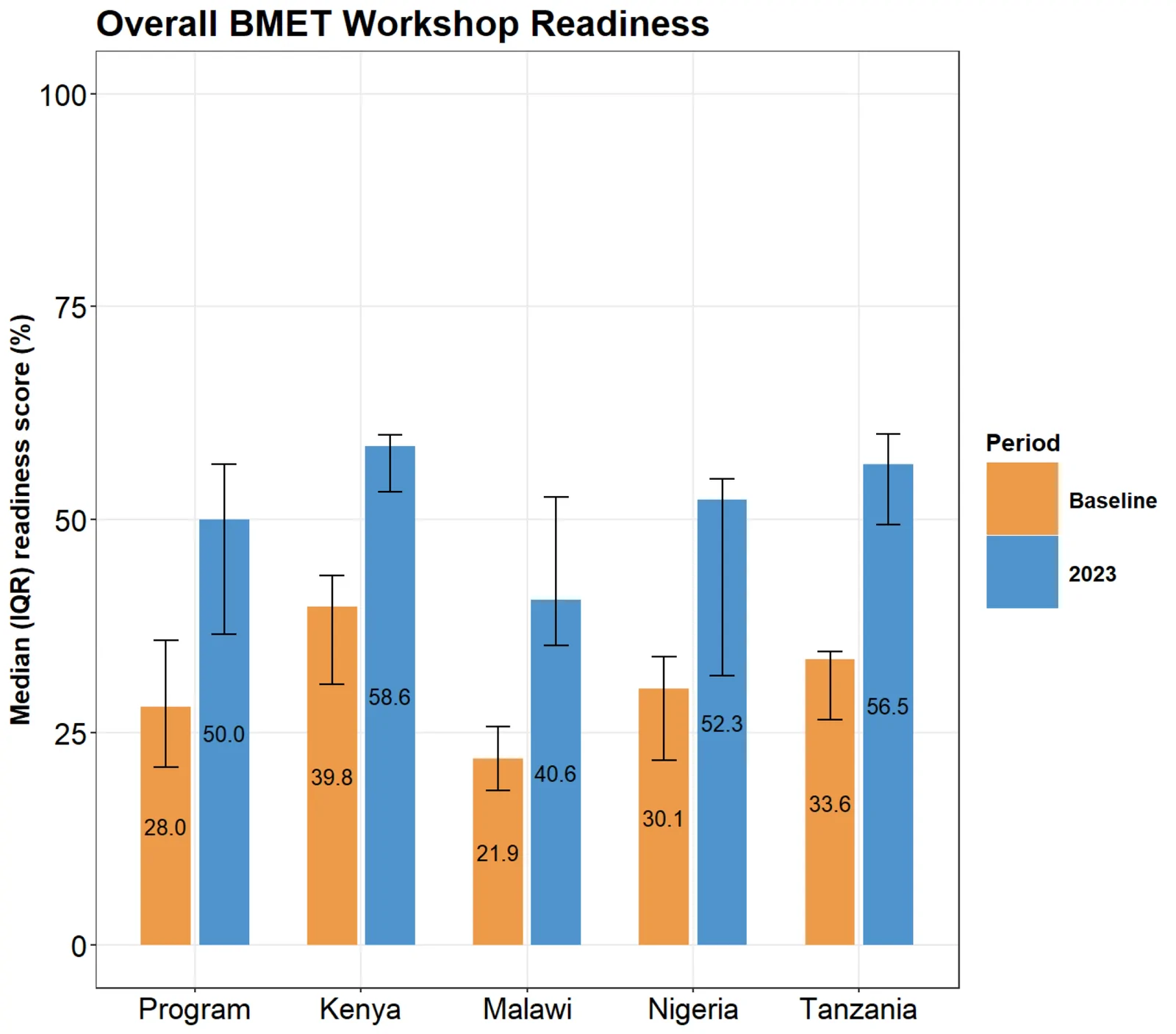
Overall programme and country-level readiness of BMET workshops in 2020–2021 (baseline) and in 2023.

Median BMET workshop readiness at baseline across 53 hospitals was 28.0% (IQR = 20.9–35.8), increasing to 50.0% (IQR = 36.5–56.5) in the 2023 cross-sectional sample of 63 hospitals (**Figure 1**).

Country-level patterns showed consistently higher readiness in the 2023 cross-sectional sample compared with baseline across all four countries. At baseline, readiness was highest in Kenya (Mdn = 39.8%, IQR = 30.6–43.4), followed by Tanzania (Mdn = 33.6%, IQR = 26.5–34.5), Nigeria (Mdn = 30.1%, IQR = 21.8–33.9), and Malawi (Mdn = 21.9%, IQR = 18.2–25.7). In 2023, Median readiness increased to 58.6% (IQR = 54.3–59.8) in Kenya, 56.5% (IQR = 49.4–60.0) in Tanzania, 52.3% (IQR = 31.7–54.8) in Nigeria, and 39.6% (IQR = 35.0–52.0) in Malawi. Sensitivity analyses restricted to a matched subset of hospitals assessed at both time points showed similar patterns across domains and countries, supporting the robustness of the observed improvements despite differences in hospital composition between assessment rounds (Table S3 in the **Online Supplementary Document**). Overall BMET workshop readiness was significantly higher in the 2023 sample than at baseline (Wilcoxon rank-sum test: W = 497, *P* < 0.001), indicating a shift towards higher hospital-level readiness across participating hospitals.

We observed the largest improvements in median hospital-level readiness between baseline and 2023 in the availability of tools, increasing from 13.5% (IQR = 3.0–21.6) to 62.2% (IQR = 45.9–74.3), and in maintenance and repair capacity, increasing from 52.9% (IQR = 35.3–76.5) to 94.1% (IQR = 76.5–100). In contrast, human resources showed relatively stable readiness, with little change between baseline (58.3%, IQR = 41.7–66.7) and 2023 (62.5%, IQR = 41.7–68.8) across the programme (**Figure 1**).

### Availability of tools

**Figure 2:**
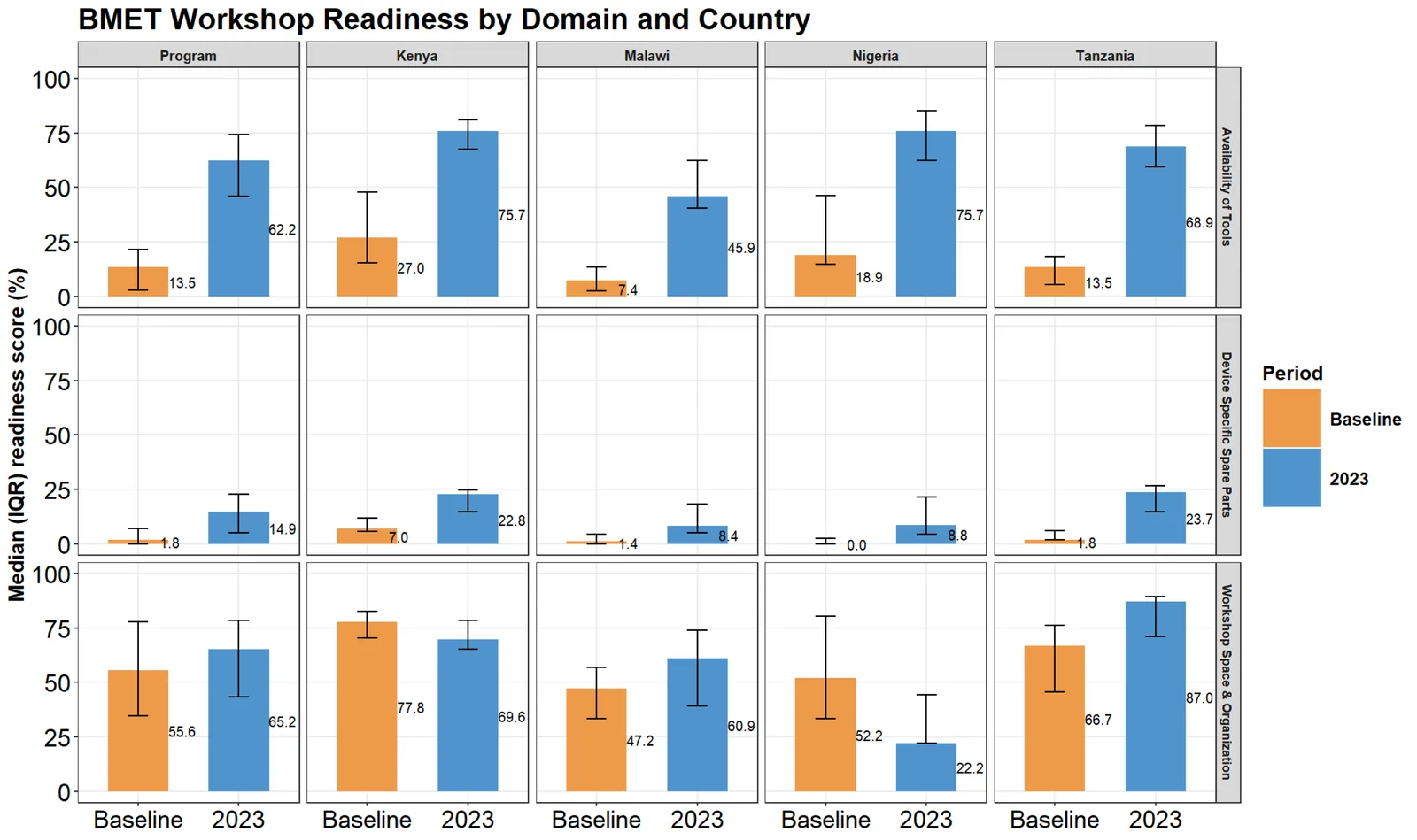
Overall programme and country-level availability of tools, device-specific spare parts, and readiness of biomedical workshop space, storage and organisation for BMET workshops in 2020–2021 (baseline) and in 2023.

Median tool availability across hospitals increased substantially from 13.5% (IQR = 3.0–21.6) at baseline to 62.2% (IQR = 45.9–74.3) in 2023 (**Figure 2**). This pattern was consistent across all countries, with the largest gains observed in Tanzania, where median availability increased from 13.5% (IQR = 5.4–18.2) to 68.9% (IQR = 59.5–78.4).

Hospital-level tool availability was significantly higher in 2023 than at baseline (Wilcoxon rank-sum test: W = 3039.5, *P* < 0.001), indicating a substantial shift toward improved tool availability across the programme.

Among hospitals with data at both time points, a small number showed lower tool availability in 2023 compared with baseline (one in Nigeria and two in Kenya).

The most commonly available tools in 2023 included assorted Torx screwdrivers (92.2%), snap-off blade knives (92.2%), tape measures (92.2%), and digital multimeters with accessories (89.1%). In contrast, the least available tools were penlight torches (28.1%), solder braid wicks (21.9%), bevelled cutters (20.3%), and polycarbonate brass trimmer sets (14.1%).

### Availability of device-specific spare parts

We assessed the availability of 56 device-specific spare parts across 11 types of medical devices, including pulse oximeters, radiant warmers, incubators, oxygen cylinders, oxygen concentrators, oxygen flow splitters, electric suction pumps, manual suction pumps, light-emitting diode and fluorescent phototherapy devices, and CPAP drivers (Table S1 in the **Online Supplementary Document**).

Hospital-level spare parts availability was low at baseline, with a median of 1.8% (IQR = 0.0–7.0), increased to 14.9% (IQR = 5.3–22.8) in 2023 (**Figure 2**). We observed improvements across all countries, with median availability in 2023 reaching 22.8% (IQR = 14.9–24.6) in Kenya, 23.7% (IQR = 14.9–26.8) in Tanzania, 8.8% (IQR = 4.4–21.4) in Nigeria, and 8.4% (IQR = 5.1–18.4) in Malawi.

Hospital-level spare parts availability was significantly higher in 2023 than at baseline (Wilcoxon rank-sum test: W = 2743.5, *P* < 0.001), although distributions remained wide, with some hospitals reporting no availability.

In 2023, the most readily available spare parts were for pulse oximeters (Mdn = 33.3%, IQR = 0.0–66.7) and oxygen cylinders (Mdn = 33.3%, IQR = 0.0–66.7), followed by electric suction pumps (Mdn = 16.7%, IQR = 0.0–16.7%) and oxygen concentrators (Mdn = 12.5%, IQR = 6.2–18.8). Spare parts availability for other device categories remained minimal, with median availability of 0.0% (Figure S4 in the **Online Supplementary Document**).

### Workshop space, storage and organisation

We assessed the workshop space, storage, and organisation domain using 23 indicators, including availability of independent workspaces, device sorting systems, storage for new equipment, fire extinguishers, record-keeping systems, quality assurance measures, adequate lighting, and loading/unloading areas (Table S1 in the **Online Supplementary Document**).

At both time points, nearly all hospitals reported having independent BMET workspaces (96.3% at baseline and 98.4% in 2023).

Median hospital-level readiness for this domain was 55.6% (IQR = 34.8–77.8) at baseline and 65.2% (IQR = 43.5–78.3) in 2023 across the programme (**Figure 2**).

Country-level patterns were mixed; Tanzania showed a marked increase from 66.7% (IQR = 45.6–76.1) to 87.0% (IQR = 71.0–89.2), and Malawi also improved from 47.2% (IQR = 33.3–56.9) to 60.9% (IQR = 39.1–73.9). In contrast, we observed lower readiness in 2023 than at baseline in Kenya (Mdn = 77.8%, IQR = 70.3–82.6 to Mdn = 69.6%, IQR = 65.2–78.3) and Nigeria (Mdn = 52.2%, IQR = 33.3–80.4 to MD = 22.2%, IQR = 22.2–44.5).

Overall, the difference in hospital-level readiness between baseline and 2023 was not statistically significant (Wilcoxon rank-sum test: W = 1838, *P* = 0.351).

### BMET systems and processes

Effective systems and processes, including systems for PPM, CM, procurement planning, and quality improvement initiatives, are essential for maintaining medical devices and ensuring continuous readiness for patient care.

At baseline, 68.5% of hospitals reported having a PPM system in place, compared with 90.6% in the 2023 cross-sectional sample, indicating a higher proportion of hospitals with established PPM systems at follow-up. Notably, the six hospitals without PPM systems in 2023 were all located in Malawi. In contrast, the proportion of hospitals reporting CM systems was similar at baseline (75.9%) and in 2023 (76.6%), indicating little change between assessment rounds.

**Figure 3:**
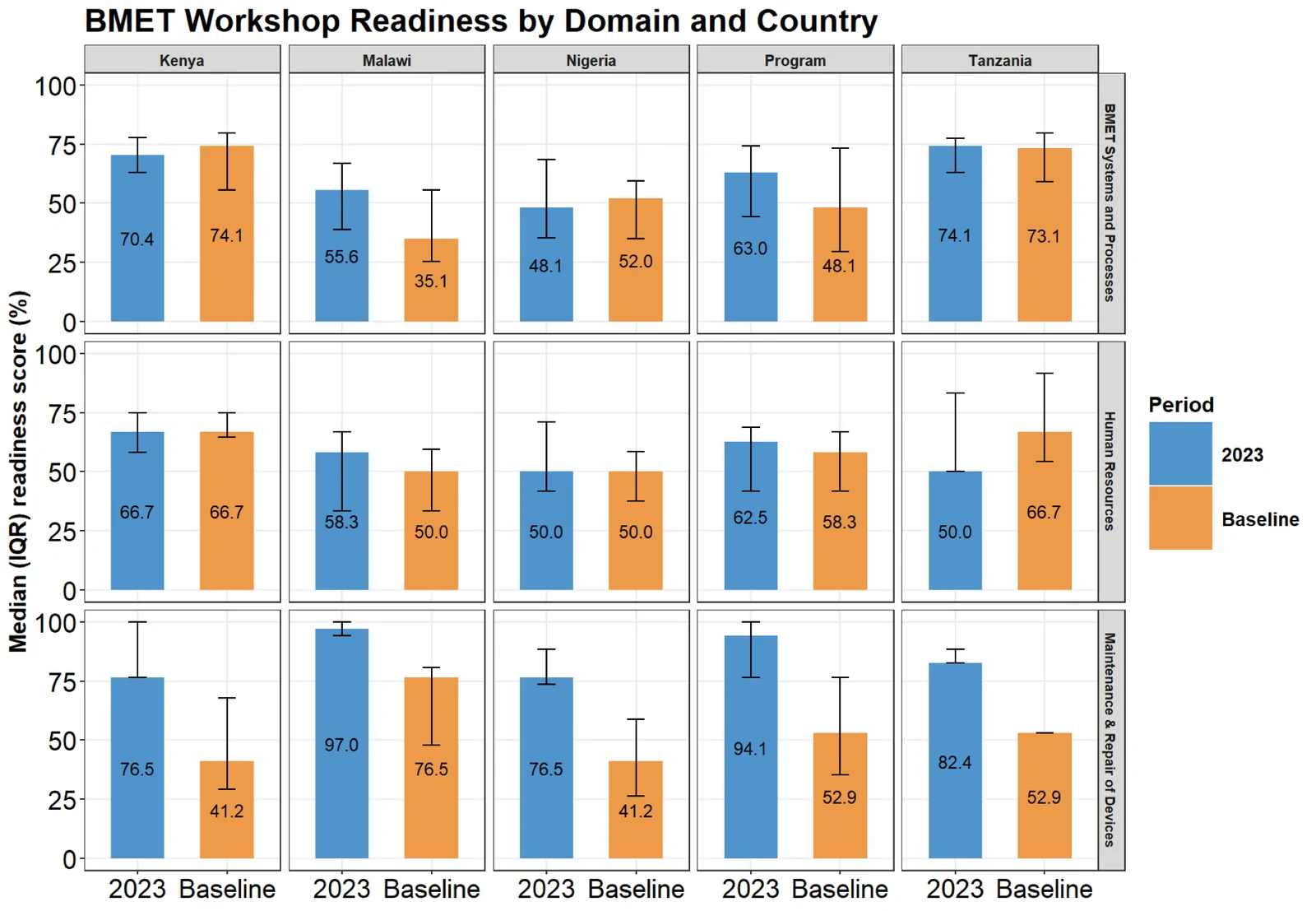
Overall programme and country-level BMET workshop readiness scores for BMET systems and processes, human resources, and maintenance and repair of medical devices in 2020–2021 (baseline) and in 2023.

Across the 27 indicators in this domain, median hospital-level readiness was 48.1% (IQR = 29.6–73.1) at baseline and 63.0% (IQR = 44.4–74.1) in 2023 (**Figure 3**). Country-level patterns were mixed; Malawi showed higher readiness in 2023 (Mdn = 55.6%, IQR = 38.9–66.7) compared with baseline (Mdn = 35.1%, IQR = 25.4–55.6), while Tanzania showed similar levels across both time points (Mdn = 73.1%, IQR = 59.2–79.7 at baseline and Mdn = 74.1%, IQR = 63.0–77.4 in 2023). In contrast, we observed slightly lower median readiness in 2023 in Kenya and Nigeria.

Overall, the difference in hospital-level readiness between baseline and 2023 was not statistically significant (Wilcoxon rank-sum test: W = 1975, *P* = 0.091).

### Biomedical workshop staffing and allocation to hospitals

Hospital-level readiness for the human resources domain was similar between baseline (Mdn = 58.3%, IQR = 41.7–66.7) and 2023 (Mdn = 62.5%, IQR = 41.7–68.8) across the programme (**Figure 3**). Country-level patterns showed minimal variation between time points, with largely overlapping distributions across all four countries.

Overall, there was no statistically significant difference in hospital-level human resource readiness between baseline and 2023 (Wilcoxon rank-sum test: W = 1815.5, *P* = 0.416).

These scores reflect hospital-level staffing availability and do not capture temporal coverage (*e.g.* night or weekend shifts) or the allocation of personnel specifically to neonatal unit BMET workshop activities. As such, they may overestimate functional staffing capacity for continuous maintenance and repair services.

### Maintenance and repair of devices

Median hospital-level capacity to maintain and repair CPAP machines, phototherapy devices, and oxygen concentrators was substantially higher in 2023 than at baseline, increasing from 52.9% (IQR = 35.3–76.5) to 94.1% (IQR = 76.5–100) across the program (**Figure 3**).

We observed improvements were observed across all countries, with median readiness increasing from 41.2% (IQR = 29.4–67.7) to 76.5% (IQR = 76.5–100) in Kenya, from 76.5% (IQR = 47.8–80.6) to 97.0% (IQR = 94.1–100) in Malawi, from 41.2% (IQR = 26.4–58.8) to 76.5% (IQR = 73.6–88.2) in Nigeria, and from 52.9% (IQR = 52.9–52.9) to 82.4% (IQR = 82.4–88.2) in Tanzania.

Overall, hospital-level maintenance and repair capacity was significantly higher in 2023 than at baseline (Wilcoxon rank-sum test: W = 515.5, *P* < 0.001), indicating a substantial shift toward improved readiness across hospitals.

Since we interviewed only one staff member per hospital, these findings reflect reported capacity and may not capture variation across staff within hospitals. This may particularly influence results in settings with small biomedical teams (*e.g.* Malawi) and could limit generalisability to hospitals with larger or more specialised staffing structures.

### Documentation tools for BMET workshop operations

Documentation tools are essential for enabling accountability, planning, and performance monitoring within BMET workshops. Across the four countries, several documentation indicators were more commonly reported in the 2023 sample than at baseline.

In Malawi, the proportion of hospitals reporting use of PPM job cards were substantially higher in 2023 (80.6%) compared with baseline (24.1%). Similarly, the use of CM job cards increased from 69.0% at baseline to 86.1% in 2023.

In Nigeria and Tanzania, both CM and PPM job card use were reported in all hospitals in the 2023 sample. However, given small sample sizes and variability at baseline, these patterns should be interpreted with caution. In Kenya, CM job card use was slightly higher in 2023 (92.3%) compared with baseline (81.8%), while PPM job card use remained consistently high across both time points (81.8% at baseline and 84.6% in 2023), indicating little change.

Other documentation systems showed similar patterns. The proportion of hospitals reporting use of stock cards was higher in 2023 in Kenya and Malawi, and availability of tool requisition registers increased in Malawi (from 20.7% to 52.8%). In contrast, some documentation practices, such as decommissioning records and equipment tagging systems, remained uncommon across several countries, with limited change between assessment rounds (Figure S14 in the **Online Supplementary Document**).

### Allocation of biomedical human resources to hospitals

The availability and allocation of biomedical engineers, biomedical technicians, and electricians – representing hospital-wide staffing capacity rather than BMET workshop-specific deployment – varied across countries and between assessment periods. Across all countries, the Ministry of Health remained the primary employer of biomedical technicians (Kenya 92%, Malawi 78%, Nigeria 63%, Tanzania 80%).

Kenya showed a shift in workforce composition by 2023, with fewer biomedical engineers per hospital (Mdn = 1, IQR = 1–2) compared with baseline (Mdn = 5, IQR = 2–9), alongside a higher number of biomedical technicians (Mdn = 7, IQR = 5–9 *vs.* Mdn = 2, IQR = 1–6). Malawi showed little change, maintaining a median of one biomedical engineer (IQR = 1–1) and one biomedical technician (IQR = 1–2) per hospital across both time points. Nigeria and Tanzania showed mixed patterns: Nigeria reported fewer biomedical technicians in 2023 (Mdn = 4, IQR = 2–4 *vs.* Mdn = 6, IQR = 2–60), while Tanzania showed higher technician availability (Mdn = 5, IQR = 2–6 *vs.* Mdn = 3, IQR = 3–5).

The number of electricians attached to BMET workshops was higher in 2023 in Kenya (Mdn = 2 *vs.* Mdn = 1) and Tanzania (Mdn = 3 *vs.* Mdn = 2), remained unchanged in Malawi (Mdn = 1), and was slightly lower in Nigeria (Mdn = 3 *vs.* Mdn = 4) (Figure S2 in the **Online Supplementary Document**).

Day-shift coverage remained the strongest across all settings. In 2023, Kenya and Tanzania reported the highest number of personnel physically present during weekday shifts (both Mdn = 5; IQR = 4–9 and IQR = 3–11, respectively). Night and weekend coverage remained limited, with median staffing of zero (IQR = 0–1) across most countries. On-call availability during night shifts was slightly higher in 2023 in Kenya and Malawi (Mdn = 1, IQR = 0–1) compared with baseline (Mdn = 0, IQR = 0–1).

Overall, despite shifts in workforce composition in some countries, gaps in round-the-clock staffing persisted across all settings.

### Maintenance and repair of medical devices

Maintenance and repair of medical devices varied across hospitals and between assessment rounds in the four countries. Equipment inventory lists were available in 100% of hospitals in Tanzania in 2023, compared with 83% at baseline. In Malawi, inventory availability was higher in 2023 (67%) than at baseline (43%), while Nigeria had slightly lower availability (75% in 2023 *vs.* 86% at baseline).

While most hospitals continued to offer free repair services, the proportion was lower in 2023 in Kenya (85% *vs.* 100% at baseline) and Tanzania (86% *vs.* 100% at baseline); however, especially for Kenya, this may reflect differences in the hospitals included at each time point rather than a true decline. Funding for parts relied largely on hospital revenues, with minimal external or government support. Persistent spare parts shortages were reported as the major barrier to repairing medical devices in 69% of Kenyan, 78% of Malawian, 100% of Nigerian, and 71% of Tanzanian hospitals. Other reported barriers included insufficient technicians and inadequate training (**Table 1**).

**Table 1:** Maintenance and repair of medical devices at NEST360 implementing hospitals*

	Kenya		Malawi		Nigeria		Tanzania	
**Characteristics**	**2020–2021 (baseline), N = 11**	**2023, N = 13**	**2020–2021 (baseline), N = 28**	**2023, N = 36**	**2020–2021 (baseline), N = 7**	**2023, N = 7**	**2020–2021 (baseline), N = 7**	**2023, N = 7**
**Equipment inventory**	11/11 (100)	10/13 (77)	12/27 (44)	24/36 (67)	6/7 (86)	5/7 (71)	5/6 (83)	7/7 (100)
**Biomedical technician’s employer**								
Hospital	0/11 (0)	1/12 (8.3)	8/28 (29)	8/36 (22)	3/7 (43)	2/7 (29)	4/6 (67)	1/5 (20)
Ministry of Health	11/11 (100)	11/12 (92)	19/28 (68)	28/36 (78)	4/7 (57)	5/7 (71)	1/6 (17)	4/5 (80)
Don’t know			1/28 (3.6)	0/36 (0)			1/6 (17)	0/5 (0)
**Free repair of medical equipment**	11/11 (100)	11/13 (85)	22/28 (79)	36/36 (100)	5/7 (71)	5/7 (71)	6/6 (100)	6/7 (86)
**Primary funder for spare parts**								
Donation from NGO	0/11 (0)	1/13 (7.7)	1/28 (3.6)	2/36 (5.6)				
Government	2/11 (18)	4/13 (31)	3/28 (11)	2/36 (5.6)	0/7 (0)	1/7 (14)	0/6 (0)	1/7 (14)
Hospital	9/11 (82)	8/13 (62)	24/28 (86)	32/36 (89)	7/7 (100)	5/7 (71)	6/6 (100)	3/7 (43)
Don’t know					0/7 (0)	1/7 (14)		
Other							0/6 (0)	3/7 (43)
**Biggest reported barrier to repairing equipment**								
Cost of repair	1/11 (9.1)	2/13 (15)					0/6 (0)	1/7 (14)
Insufficient number of technicians	0/11 (0)	2/13 (15)	4/28 (14)	3/36 (8.3)	2/7 (29)	0/7 (0)	1/6 (17)	0/7 (0)
Lack of spare parts	8/11 (73)	9/13 (69)	16/28 (57)	28/36 (78)	4/7 (57)	7/7 (100)	2/6 (33%)	5/7 (71)
Lack of training for technician	2/11 (18)	0/13 (0)	4/28 (14)	5/36 (14)			2/6 (33)	0/7 (0)
Lack of tools for repair			3/28 (11)	0/36 (0)				
Other			1/28 (3.6)	0/36 (0)	1/7 (14)	0/7 (0)	1/6 (17)	1/7 (14)

NGO – non-governmental organisation

*Presented as n/N (%). Proportions calculated based on available data.

### PPM frequency and BMET readiness

Programme-wide PPM completion rates were higher in the later reporting period, increasing from an average of 75% in 2022 to 90% in 2023 (Figure S3, Panel A in the **Online Supplementary Document**). We observed the largest increases in Kenya (49% to 85%) and Tanzania (41% to 94%), while Malawi maintained consistently high completion rates across periods. Nigeria showed similarly high completion rates across both periods.

Exploratory hospital-level analyses assessed the relationship between BMET workshop readiness scores measured in 2023 and mean PPM completion rates during the same period. Across 63 hospitals, there was little evidence of a strong correlation between BMET readiness and PPM completion (Spearman’s rho = 0.11, *P* = 0.375) (Figure S3, Panel B in the **Online Supplementary Document**). We observed substantial variability in PPM completion across hospitals with similar readiness scores, suggesting that factors beyond measured BMET workshop readiness may influence PPM implementation.

### Device functionality and BMET workshop readiness

BMET workshop readiness scores were positively associated with device functionality at baseline (r = 0.32, *P* = 0.018), indicating that higher workshop readiness co-occurred with better equipment functionality in this cross-sectional sample. This association was strongest in Kenya (r = 0.65, *P* = 0.031), while a similar but non-significant pattern was observed in Tanzania (r = 0.65, *P* = 0.11).

In the 2023 cross-sectional sample, we observed no statistically significant associations between BMET workshop readiness and device functionality overall (r = –0.10, *P* = 0.43), and no country-level correlations reached statistical significance. These findings suggest that the relationship between workshop readiness and device functionality was not consistently observed across assessment periods.

## DISCUSSION

This evaluation of BMET workshops across four sub-Saharan African countries demonstrates measurable progress in health technology management during the NEST360 Phase 1 implementation. At the hospital level, overall BMET workshop readiness increased between assessment rounds, with median scores rising from 28.0% (IQR = 20.9–35.8) at baseline to 50.0% (IQR = 36.5–56.5) in 2023.

Across domains, improvements were most pronounced in tool availability and maintenance and repair capacity, while gains in human resources were modest. Spare parts availability also increased over time, although levels remained low overall. Despite these improvements, persistent shortages of spare parts were consistently reported as the major barrier to timely repair across countries. Most hospitals continued to rely on internal budgets for repair activities, with limited external financing and weak integration of spare parts into central procurement systems.

Workforce constraints also persisted across both time points. Biomedical engineering capacity remained largely concentrated in weekday daytime operations, with limited night and weekend coverage across all settings. This indicates that despite improvements in physical resources and documented systems, service delivery remains constrained by staffing models that do not support continuous maintenance needs.

The marked increase in essential tool availability in BMET workshops likely reflects targeted investments in equipping workshops for routine maintenance and emergency repairs, alongside ongoing engagement with Ministries of Health in participating countries. However, the decline in tool availability observed in a small number of hospitals (two in Kenya and one in Nigeria) suggests that progress was not uniform and may reflect hospital-level differences, procurement practices, or local supply chain constraints rather than a systematic reversal of gains. A study of district hospitals in Rwanda similarly reported that while basic tools were generally available, biomedical technicians often lacked access to specialised equipment, limiting their ability to complete certain repairs [16]. This highlights persistent gaps in the availability of task-specific tools required for effective biomedical maintenance. The WHO has similarly emphasised that biomedical engineering productivity is constrained in the absence of appropriate tools and test equipment [17]. Ensuring adequate provision of such resources remains important for supporting timely, safe, and effective device maintenance and reducing equipment downtime.

Although spare parts were the most frequently reported barrier to biomedical equipment repair, their availability increased between assessment periods, representing an important improvement in maintenance readiness. While NEST360 provided spare parts for donated equipment, the extent to which this contributed to overall improvements is unclear, as data on hospital-level procurement and third-party supply chains were not available. Improvements were particularly notable in Tanzania, likely reflecting broader investments by multiple stakeholders during and after the COVID-19 pandemic to strengthen oxygen infrastructure [18]. However, despite these gains, availability of specialised and device-specific spare parts remained low across settings, with more basic components such as oxygen tubing, fuses, and bulbs more consistently available, suggesting a persistent gap in higher-order technical components required for full device functionality. The WHO has emphasised that limited access to replacement parts undermines maintenance systems and contributes to prolonged equipment downtime [17], and Perry and Malkin similarly highlighted that weak procurement systems and limited integration of spare parts planning into maintenance workflows constrain BMET workshop effectiveness [5]. Strengthening supply chain systems and institutionalising dedicated budgets for spare parts therefore remain critical for sustaining maintenance improvements in low-resource settings, where manufacturer-supported servicing is often limited [19].

The findings from the maintenance and repair assessments across the four countries demonstrate both progress and persistent challenges in ensuring medical device functionality. We observed improvements in equipment inventory systems in some settings, with Tanzania reporting near-universal availability of inventory lists and Malawi showing marked increases in inventory availability over time. However, the availability of free repair services was inconsistent across all hospitals or time points, highlighting variability in maintenance service provision and potential sustainability concerns linked to resource constraints. These challenges are particularly relevant in public hospital settings, where constrained budgets and centralised supply chain processes may limit timely repair and maintenance of equipment [20].

The Ministry of Health’s role as the primary employer of biomedical technicians across countries indicates an important structural foundation for biomedical engineering capacity. However, variability in technician availability, distribution, and training across settings suggests that staffing alone does not fully translate into consistent maintenance coverage or service delivery capacity. These findings point to broader health system constraints affecting the sustainability of biomedical maintenance services, particularly in settings reliant on donated equipment and constrained fiscal space. Strengthening maintenance systems may therefore require improved integration of biomedical services into routine hospital planning and budgeting processes, alongside continued investment in technical training, inventory management, and structured maintenance systems. Ensuring that medical equipment remains functional and responsive to clinical needs will be particularly important in low-resource settings where external support and supply chains are variable [21,22].

Human resources’ readiness showed little change between baseline and 2023, with no statistically significant differences and notable variability across countries. Biomedical technicians were consistently reported as primarily employed by Ministries of Health, reflecting a shared structural model for workforce deployment across settings. However, staffing patterns suggest a predominance of routine daytime coverage with limited night and weekend availability, indicating a persistent gap in 24/7 maintenance support. Although human resources median scores (~58–62%) capture staffing levels and institutional allocation, they do not fully reflect temporal coverage of services. In contrast, hospital-level evidence of limited after-hours staffing highlights a critical dimension of service readiness not captured by the composite score. Together, these findings suggest that biomedical workforce adequacy depends not only on staffing levels but also on deployment patterns, particularly shift coverage and after-hours responsiveness, especially in these settings where more than 50% of devices may be defective at any given time [21].

Beyond hospital-level staffing, sustained investment in biomedical engineering training pipelines remains important for ensuring a sufficiently skilled and distributed workforce over time [23,24]. Biomedical personnel are central to the lifecycle of medical devices and essential for sustaining health system functionality [25]. Evidence from Rwanda further demonstrates the importance of trained biomedical staff, where hospitals with engineering WHO-trained technicians showed lower device downtime and higher corrective maintenance activity compared with those without trained personnel [26]. Together, these findings highlight that both adequate staffing structures and targeted technical training are necessary to support functional and responsive biomedical maintenance systems.

The observed association between BMET workshop readiness and device functionality at baseline suggests that biomedical capacity was linked to equipment performance in the earlier period, particularly in settings such as Kenya and Tanzania where correlations were strongest. However, we did not observe this relationship in the 2023 cross-sectional sample, indicating a potential decoupling between workshop readiness and device functionality over time. This shift may reflect broader changes in the health system during the implementation period, including improvements in maintenance practices, training, and preventive maintenance systems, as well as potential changes in the composition or functionality of equipment across hospitals. However, given the cross-sectional nature of the data, these explanations remain exploratory. Despite the absence of this relationship in 2023, important system constraints persisted, particularly in relation to spare parts availability and staffing coverage, which were consistently identified as limiting factors across domains. Strengthening supply chains and ensuring reliable biomedical workforce support therefore remain important for translating workshop capacity into sustained improvements in device functionality and service reliability.

### Strengths and limitations

This study has several strengths. First, it used a repeated cross-sectional design with assessments conducted at two time points (2019–2021 and 2023), enabling comparison of BMET workshop readiness across two independent samples of hospitals over time within the context of NEST360 programme implementation. Rather than tracking the same hospitals longitudinally, the design provides population-level snapshots of biomedical engineering readiness at two distinct periods. Second, the study included a wide range of hospital types, from district hospitals to national referral centres, enhancing the contextual relevance and generalisability of findings across different levels of the health system. Third, the multi-country scope across four sub-Saharan African countries supports regional comparison and highlights both shared and context-specific patterns in biomedical workshop readiness.

In addition, the study applied a comprehensive, multi-domain framework covering key components of biomedical engineering workshop functionality, including tools and equipment, spare parts availability, human resources, maintenance systems, and documentation processes. This provides a holistic assessment of the structural components required to support biomedical equipment maintenance.

Several limitations should also be acknowledged. Because the baseline and 2023 samples include different sets of hospitals, observed differences between time points reflect both temporal change and variation in hospital composition. As a result, findings should be interpreted as comparisons between cross-sectional samples rather than within-hospital longitudinal changes. In addition, the scoring framework applied equal weighting across indicators within domains, which may not fully reflect the relative importance of specific items for functional biomedical service delivery. Alternative weighting approaches could potentially yield different composite estimates of readiness.

Furthermore, although we designed the assessment to capture BMET workshop readiness for hospital-wide biomedical needs, we weighted some indicators toward neonatal care settings and neonatal device-specific systems. This may limit the generalisability of certain findings to other clinical departments within hospitals. We also relied primarily on a single key informant per hospital for the BMET module, typically the most senior available biomedical technician, which may introduce reporting bias and limit the capture of variability in knowledge, practice, and workshop conditions where multiple biomedical staff operate within the same hospital, particularly in larger hospitals. Finally, while our study captures structural readiness across multiple domains, it does not directly measure functional outcomes such as device downtime, repair turnaround time, or service interruption duration, which limits inference on operational impact.

## CONCLUSIONS

We found that BMET workshop readiness improved between baseline and 2023 across multiple domains in repeated cross-sectional samples from four sub-Saharan African countries in the context of NEST360. These improvements were accompanied by consistently higher median readiness scores in most domains, alongside statistically significant differences in several key components of biomedical workshop readiness.

Despite these gains, important gaps persisted across the health system. Human resource readiness showed minimal change over time, and staffing coverage remained largely limited to daytime operations, with persistent gaps in night and weekend support. In addition, spare parts availability, while improved, remained constrained for more specialised device components, and variability across countries and hospitals was substantial.

Overall, these findings suggest that improvements in biomedical workshop readiness are occurring alongside persistent structural constraints in workforce deployment and supply chain integration. Sustaining and consolidating these gains will likely require continued strengthening of biomedical engineering systems, including improved integration of maintenance needs into procurement and budgeting processes and more consistent institutional support for biomedical services. For policymakers and programme implementers, this underscores the importance of embedding biomedical engineering functions within routine health system planning, supported by stable supply chains and appropriately deployed human resources.

## Additional material


Online Supplementary Document

## Data Availability

**Data availability:** Data sharing and transfer agreements were jointly developed and signed by all collaborating partners in the NEST360 alliance. The HFA tool, data dictionary, and associated materials are available from the NEST360/UNICEF Implementation Toolkit for Small and Sick Newborn Care (https://newborntoolkit.org/tools/nest360-health-facility-assessment-hfa?language=en) and the NEST360 website (https://nest360.org/project/hfa/).
