# Supplementary material for: Strengthening biomedical engineering systems in sub-Saharan Africa: a repeated cross-sectional assessment of workshop readiness and medical device maintenance in NEST360-supported hospitals
Source: J Glob Health. 2026 Sep 25;16:04270. doi: 10.7189/jogh.16.04270 (PMC13613297; doi:10.7189/jogh.16.04270)
Supplement: Supplementary file 1 [file jogh-16-04270-s001.pdf]

Supplement to: Wainaina J, Bohne C, Banda G, Ochieng V, Osuagwu C, Ngowi E, Peter H, Jenkins G, Madete J, Alooh M, Nambiar A, Liaghati S, Ogero M, Penzias R, Kumara D, Hirschhorn L, Oden Z, Richards-Kortum R. Strengthening biomedical engineering systems in sub-Saharan Africa: a repeated cross-sectional assessment of workshop readiness and medical device maintenance in NEST360-supported hospitals. J Glob Health. 2026;16:04270.

SUPPLEMENTARY MATERIALS

Table S1: List of indicators scored per category

Spare Parts and Tools

Sub-Category	Indicators
PPEs	Overall/jumpsuits, reusable gloves, dust/debris face masks, surgical face masks, N95 face masks, face shields, infection prevention goggles, safety goggles…
General Spares/Tools	Power cables, Batteries 9V, Batteries AA, Batteries AAA, Fuses …
Device specific spares	Assorted list of spares for:<br><br>**Pulse Oximeters**: *Neonatal sized pulse ox' probe (reusable, clip), Neonatal sized pulse oximetry probe (reusable, wrap), Recharger for batteries*<br><br>**Radiant warmers**: *Temperature probe, Circuit board (control), Circuit board (heating element), Power supply unit, LCD*<br><br>**Incubators**: *Circuit board (main control), Heating element, Temperature probe, fan, Control panel (membrane or LCD),*<br><br>**Oxygen cylinders**: *Pressure regulator, Stop valve, cap, pressure gauge, flowmeter, Pigtail with safety wire*<br><br>**Oxygen concentrators**: *Flowmeter, Circuit board (control), Circuit board (oxygen monitoring), Circuit board/step down transformer (fan), fan, Solenoid valves (4-way), Solenoid valves (equalization), Molecular sieve beds, Power supply unit, Intake filter (internal, fine particle), Intake filter (external, gross particle), Reservoir tank, Muffler with exhaust filter, Compressor, Compressor rebuild component, Starting capacitor*<br><br>**Suction Pump (portable, electrical with accessories):** *Pump assembly, Vacuum gauge, Filter (bacterial), Collection reservoir, Collection reservoir lid, Power supply unit.*

	**Suction Pump (manual, non-electric power dependent):** *Piston assembly, Vacuum gauge, Filter (bacterial), Collection reservoir.*<br><br>**Oxygen flow splitters**: *Flowmeter*<br><br>**LED Phototherapy machines**: *Circuit board (control), Power supply unit, LED driver/circuit/module, Control panel (membrane or LCD)*<br><br>**Fluorescent Phototherapy machines**: *Fluorescent bulb, Power supply unit*<br><br>**CPAP drivers**: *Pump assembly, Filter (internal, pump), Flowmeter, O-ring, Power supply unit*
Tools	Digital Multi meter, Penlight torch piece, Pocket magnifier piece, 160 mm combination pliers, Junior hack saw, Hammer, soft-faced, Hammer, ballpein, Soldering station, Soldering wire, Soldering tip cleaner, Desoldering pump or sucker, imperial hex (Allen) key set, Tape measure; 3 meters; metal, metric piece, Flared screw driver, Straight tweezers, Diagonal cutter, Insulated inspection screw driver (165 mm) piece, Tx 8, Tx 10, Tx 15 and Tx 20 Torx …

Storage Space and Layout

Sub-Category	Indicators
Storage space (for new equipment)	Storage space for new equipment and the corresponding particulars: Pallet storage, temp'/humidity monitoring systems, fire extinguisher, Quality assurance systems, record keeping, controlled access, sufficient lighting, first aid kit, 6*6 space for orderly packing, outside space for (un)loading, ventilation, location secured …
Minimum documentation for new equipment	Date of receipt, name and description, quantity received, name address of supplier, batch numbers …
BMET workshop's space and documentation	Independent space for the workshop, device sorting system, PPM job cards, CM job cards, stock cards, tool requisition register, spare parts requisition register, equipment asset tags, decommissioning records, procurement records …

Systems and Processes

Sub-Category	Indicators
Systems and Processes	PPM systems, CM systems, equipment leasing/servicing contracts, electronic maintenance systems, procurement plan for medical devices, guidelines for equipment donation, monthly reports, involvement in facility meetings e.g., QI, procurement, budget planning etc.…
Minimum documentation for new equipment	Date of receipt, name and description, quantity received, name address of supplier, batch numbers …
PPM	Areas covered by PPM (labor ward, NBU, Postnatal), PPM frequency

Corrective Maintenance	CM in NBU, Postnatal ward, Labor ward, electronic maintenance system
Reporting systems	To hospital admin, regional/district health officers, whether infrastructure and equipment maintenance issues are included, reports available …
Procurement of medical devices	Procurement plan in place, guidelines for receiving equipment donations, BMET teams involved in procurement, budget planning, and management meetings …

Human Resources

**Sub-Category**	**Indicators**
Allocation to BMET workshop and Availability	Whether biomeds/technicians are exclusively allocated to this facility<br><br>availability:<br><br>Weekdays: physically dayshift, physically nightshift, oncall-night<br>Weekends: physically dayshift, physically nightshift, oncall-night

Maintenance and Repair

**Sub-Category**	**Indicators**
General + Capacity to repair specific devices	Equipment inventory,<br>facility has capacity to repair and maintain CPAP/Ox concentrator, Phototherapy lights? (each device has this question repeated), training done at preservice to this service, received a course on how to provide this service? received on-the-job training on how to provide this service? Provide routine maintenance on oxygen concentrator? …

Table S2: Overall and category BMET readiness scores per analysis level from 53 hospitals (baseline) and 63 hospitals (2023).

**Category**	**Country/ Level**	**Baseline % (median, IQR)**	**2023 % (median, IQR)**
**Overall BMET Workshop Readiness**	Program	28 (20.9, 35.8)	50 (35.5, 56.5)
	Kenya	39.8 (30.6, 43.4)	58.6 (54.3, 59.8)
	Malawi	21.9 (18.2, 25.7)	39.6 (35.0, 52.0)
	Nigeria	30.1 (21.8, 33.9)	52.3 (31.7, 54.8)
	Tanzania	33.6 (26.5, 34.5)	56.5 (49.4, 60.0)

**Tools & Test Equipment**	Program	13.5 (3.0, 21.6)	62.2 (45.9, 75.7)
	Kenya	27.0 (15.6, 48.0)	75.7 (67.6, 81.1)
	Malawi	7.4 (2.7, 13.5)	45.9 (39.8, 58.1)
	Nigeria	18.9 (14.8, 46.4)	75.7 (62.2, 85.2)
	Tanzania	13.5 (5.4, 18.2)	68.9 (59.5, 78.4)
**Device-specific Spare Parts**	Program	1.8 (0.0, 7.0)	14.9 (5.3, 22.8)
	Kenya	7.0 (5.7, 11.9)	22.8 (21.5, 25.0)
	Malawi	1.4 (0.0, 4.6)	7.9 (4.2, 18.4)
	Nigeria	0.0 (0.0, 2.6)	8.8 (4.4, 21.4)
	Tanzania	1.8 (1.8, 6.2)	23.7 (14.9, 26.8)
**Storage & Workshop Organization**	Program	55.6 (34.8, 77.8)	65.2 (43.5, 78.3)
	Kenya	77.8 (70.3, 82.6)	69.6 (65.2, 78.3)
	Malawi	47.2 (33.3, 56.9)	60.9 (42.4, 70.7)
	Nigeria	52.2 (33.3, 80.4)	22.2 (22.2, 44.5)
	Tanzania	66.7 (45.6, 76.1)	87.0 (71.0, 89.2)
**Systems & Processes**	Program	48.1 (29.6, 73.1)	63.0 (44.4, 70.4)
	Kenya	74.1 (55.5, 79.7)	70.4 (64.8, 75.9)
	Malawi	35.1 (25.4, 55.6)	52.0 (37.9, 66.7)
	Nigeria	52.0 (35.1, 59.3)	48.1 (35.5, 68.6)
	Tanzania	73.1 (59.2, 79.7)	74.1 (63.0, 77.4)
**Human Resources Readiness**	Program	58.3 (41.7, 66.7)	58.3 (41.7, 66.7)
	Kenya	66.7 (64.6, 75.0)	66.7 (58.3, 72.9)
	Malawi	50.0 (33.3, 59.3)	54.1 (33.3, 66.7)
	Nigeria	50.0 (37.5, 58.4)	50.0 (41.7, 70.8)
	Tanzania	66.7 (54.1, 91.7)	50.0 (50.0, 83.3)
**Maintenance & Repair Capacity**	Program	52.9 (35.3, 76.5)	94.1 (76.5, 100)
	Kenya	41.2 (29.4, 67.7)	76.5 (76.5, 97.0)
	Malawi	76.5 (47.8, 80.6)	97.0 (94.1, 100)
	Nigeria	41.2 (26.4, 58.8)	76.5 (73.6, 88.2)
	Tanzania	52.9 (52.9, 52.9)	82.4 (82.4, 88.2)

Figure S4: Device specific spare parts availability at program and country levels

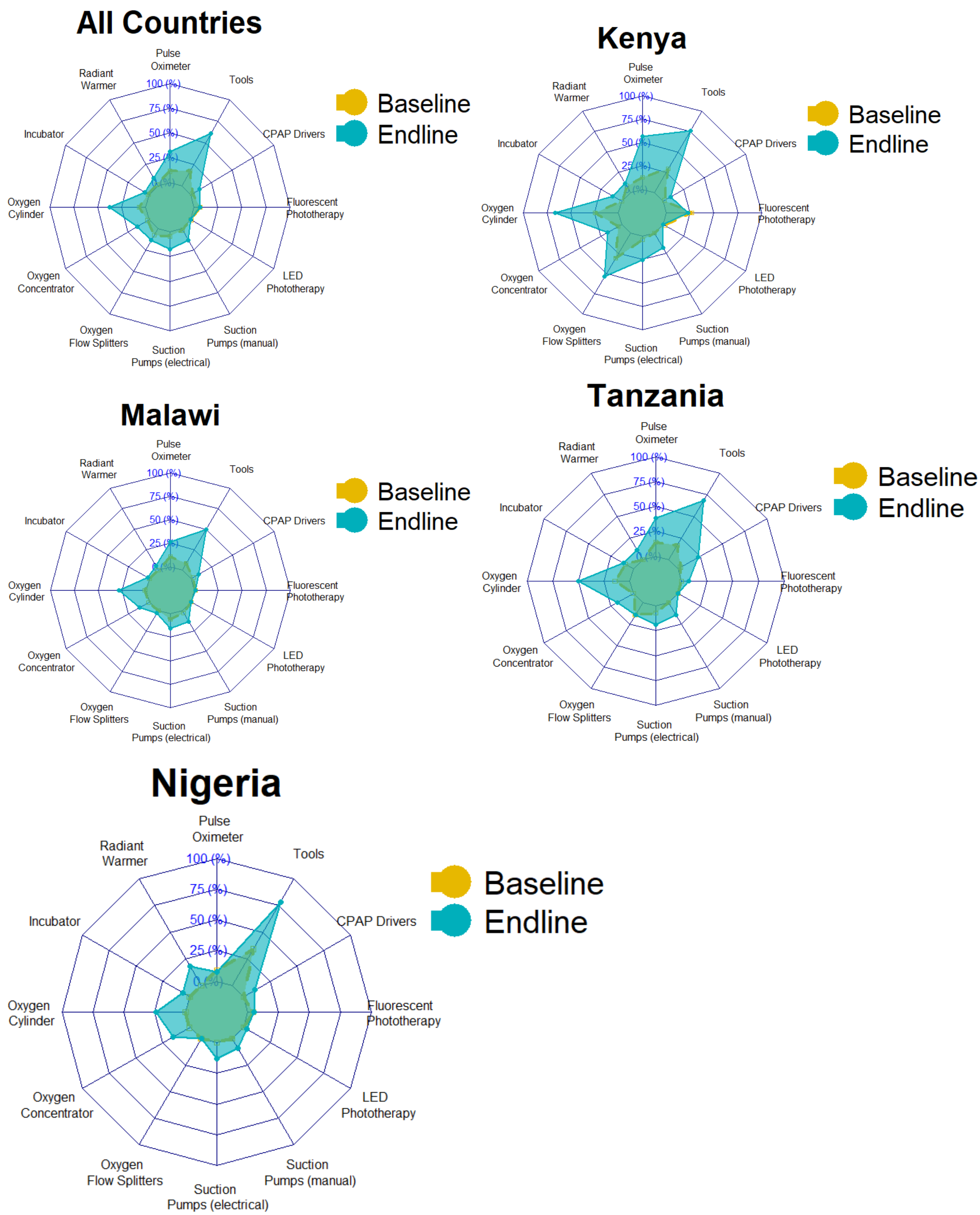

Table S3: Overall and category BMET readiness scores per analysis level, sensitivity results from 53 paired baseline and 2023 assessed hospitals

Category	Country/Level	Baseline % (median, IQR)	2023 % (median, IQR)
**Overall BMET Workshop Readiness**	Program	28.0 (20.9, 35.8)	50.0 (35.5, 56.5)
	Kenya	39.8 (30.6, 43.4)	58.6 (54.3, 59.8)
	Malawi	21.9 (18.2, 25.7)	39.6 (35.0, 52.0)
	Nigeria	30.1 (21.8, 33.9)	52.3 (31.7, 54.8)
	Tanzania	33.6 (26.5, 34.5)	56.5 (49.4, 60.0)
**Availability of Tools and Test Equipment**	Program	13.5 (3.0, 21.6)	62.2 (45.9, 75.7)
	Kenya	27.0 (15.6, 48.0)	75.7 (67.6, 81.1)
	Malawi	7.4 (2.7, 13.5)	45.9 (39.8, 58.1)
	Nigeria	18.9 (14.8, 46.4)	75.7 (62.2, 85.2)
	Tanzania	13.5 (5.4, 18.2)	68.9 (59.5, 78.4)
**Availability of Device-Specific Spare Parts**	Program	1.8 (0.0, 7.0)	14.9 (5.3, 22.8)
	Kenya	7.0 (5.7, 11.9)	22.8 (21.5, 25.0)
	Malawi	1.4 (0.0, 4.6)	7.9 (4.2, 18.4)
	Nigeria	0.0 (0.0, 2.6)	8.8 (4.4, 21.4)
	Tanzania	1.8 (1.8, 6.2)	23.7 (14.9, 26.8)
**Workshop Space, Storage and Organization**	Program	55.6 (34.8, 77.8)	65.2 (43.5, 78.3)
	Kenya	77.8 (70.3, 82.6)	69.6 (65.2, 78.3)
	Malawi	47.2 (33.3, 56.8)	60.9 (42.4, 70.7)
	Nigeria	52.2 (33.3, 80.4)	22.2 (22.2, 44.4)
	Tanzania	66.7 (45.6, 76.1)	87.0 (71.0, 89.2)
**BMET Workshop Systems and Processes**	Program	48.1 (29.6, 73.1)	63.0 (44.4, 70.4)
	Kenya	74.1 (55.6, 79.6)	70.4 (64.8, 75.9)
	Malawi	35.2 (25.4, 55.6)	52.0 (37.9, 66.7)
	Nigeria	52.0 (35.2, 59.3)	48.1 (35.4, 68.6)
	Tanzania	73.1 (59.2, 79.6)	74.1 (63.0, 77.4)
**BMET Workshop Human Resources Readiness**	Program	58.3 (41.7, 66.7)	58.3 (41.7, 66.7)
	Kenya	66.7 (64.6, 75.0)	66.7 (58.3, 72.9)
	Malawi	50.0 (33.3, 59.4)	54.2 (33.3, 66.7)
	Nigeria	50.0 (37.5, 58.4)	50.0 (41.7, 70.8)
	Tanzania	66.7 (54.2, 91.6)	50.0 (50.0, 83.3)
**Maintenance and Repair Capacity**	Program	52.9 (35.3, 76.5)	94.1 (76.5, 100.0)
	Kenya	41.2 (29.4, 67.6)	76.5 (76.5, 97.0)
	Malawi	76.5 (47.8, 80.6)	97.0 (94.1, 100.0)
	Nigeria	41.2 (26.4, 58.8)	76.5 (73.6, 88.2)
	Tanzania	52.9 (52.9, 52.9)	82.4 (82.4, 88.2)
